# Pennogenin 3-*O*-β-Chacotrioside Attenuates Hypertrophied Lipid Accumulation by Enhancing Mitochondrial Oxidative Capacity

**DOI:** 10.3390/ijms25052970

**Published:** 2024-03-04

**Authors:** Seungmin Yu, Hee Min Lee, Jangho Lee, Jin-Taek Hwang, Hyo-Kyoung Choi, Yu Geon Lee

**Affiliations:** 1 Personalized Diet Research Group, Korea Food Research Institute (KFRI), Wanju 55365, Republic of Korea; y.seungmin@kfri.re.kr (S.Y.); jhlee@kfri.re.kr (J.L.); jthwang@kfri.re.kr (J.-T.H.); chkyoung@kfri.re.kr (H.-K.C.); 2 Kimchi Industry Promotion Division, World Institute of Kimchi, Gwangju 61755, Republic of Korea; hmlee@wikim.re.kr

**Keywords:** lipid accumulation, 3T3-L1 adipocytes, lipogenesis, mitochondrial, oxidative respiration

## Abstract

Excessive lipid accumulation in adipocytes is a primary contributor to the development of metabolic disorders, including obesity. The consumption of bioactive compounds derived from natural sources has been recognized as being safe and effective in preventing and alleviating obesity. Therefore, we aimed to explore the antilipidemic effects of pennogenin 3-*O*-β-chacotrioside (P3C), a steroid glycoside, on hypertrophied 3T3-L1 adipocytes. Oil Red O and Nile red staining demonstrated a P3C-induced reduction in lipid droplet accumulation. Additionally, the increased expression of adipogenic and lipogenic factors, including PPARγ and C/EBPα, during the differentiation process was significantly decreased by P3C treatment at both the protein and mRNA levels. Furthermore, P3C treatment upregulated the expression of fatty acid oxidation-related genes such as PGC1α and CPT1a. Moreover, mitochondrial respiration and ATP generation increased following P3C treatment, as determined using the Seahorse XF analyzer. P3C treatment also increased the protein expression of mitochondrial oxidative phosphorylation in hypertrophied adipocytes. Our findings suggest that P3C could serve as a natural lipid-lowering agent, reducing lipogenesis and enhancing mitochondrial oxidative capacity. Therefore, P3C may be a promising candidate as a therapeutic agent for obesity-related diseases.

## 1. Introduction

Excessive lipid accumulation occurs owing to an imbalance in the expression of the genes responsible for fat oxidation and synthesis in adipocytes, resulting from imbalanced lifestyle choices, especially dietary habits [1]. Furthermore, an increased lipid accumulation can lead to cellular dysfunction in metabolism-regulating cells, including hepatocytes, contributing to the development of metabolic disorders, such as non-alcoholic fatty liver disease [2]. The expression of transcription factors, such as CCAAT/enhancer-binding protein (C/EBP) and peroxisome proliferator-activated receptor-γ (PPARγ), which promote the differentiation of adipocytes, is elevated in metabolic diseases such as obesity [3]. These transcriptional networks regulate the expression of the genes involved in fatty acid synthesis and transport, leading to triglyceride accumulation [4,5]. Additionally, adipocytes secrete a range of adipokines, including adiponectin, which can impair insulin sensitivity and lead to hyperglycemia and insulin resistance [6]. Therefore, the inhibition of lipid accumulation in adipocytes can be an effective strategy to prevent metabolic diseases such as obesity.

The dynamic energy balance of the cell depends on the nutritional availability [7]. Mitochondria, as the organelles responsible for energy homeostasis, coordinate bioenergetic and biosynthesis processes [8]. When cells are depleted of nutrients, mitochondria activate mechanisms that conserve energy by converting fatty acids into acetyl-CoA to promote metabolism and energy production [9]. Mitochondrial oxidative phosphorylation (OXPHOS) is the primary process for energy generation through fatty acid breakdown [10]. Conversely, over-nutrition can disable mitochondria from performing oxidative processes, leading to an increase in lipid production [11,12,13]. Anomalous mitochondria with a reduced OXPHOS capacity are a key characteristic of individuals with obesity [14,15]. Therefore, numerous studies have underscored the potential for enhancing mitochondrial oxidative function via dietary supplementation to mitigate the progression of metabolic diseases, such as obesity, by reducing fat accumulation [16,17,18,19].

Obesity is a prevalent chronic disease primarily initiated by the hypertrophy of adipocytes and the subsequent accumulation of fat in adipose tissue [20]. This lipid accumulation disrupts tissue function and contributes to the development of obesity-related comorbidities including hypertension, fatty liver disease, and cardiovascular disorders [21,22]. The economic impact of obesity encompasses both direct and indirect costs, including increased medical expenses and reduced socioeconomic productivity, and this burden continues to escalate as the obese population continues to grow [23]. Despite the growing prevalence of obesity, there are currently few effective therapies. Lifestyle interventions, such as dietary adjustments and exercise, offer only partial effectiveness [24]. Given that adopting healthy eating habits is considered a safe strategy for preventing obesity, there is a need to discover bioactive compounds capable of inhibiting lipid accumulation.

Pennogenin 3-*O*-β-chacotrioside (P3C) is the main bioactive compound derived from *Paris polyphylla*, a plant used in traditional folk medicine across Asian countries [25]. Despite numerous studies investigating the biological activities of *P. polyphylla* extracts, such as anti-inflammatory, anti-oxidant, and anti-cancer properties, limited information is available regarding the specific bioactivity of P3C [26]. Notably, P3C exhibited a significant inhibitory effect on lipid droplet accumulation in our screening analysis. Herein, we aimed to explore the antilipidemic effects of P3C in differentiated 3T3-L1 adipocytes by analyzing key lipogenic factors and mitochondrial oxidative function.

## 2. Results

**Figure 1:**
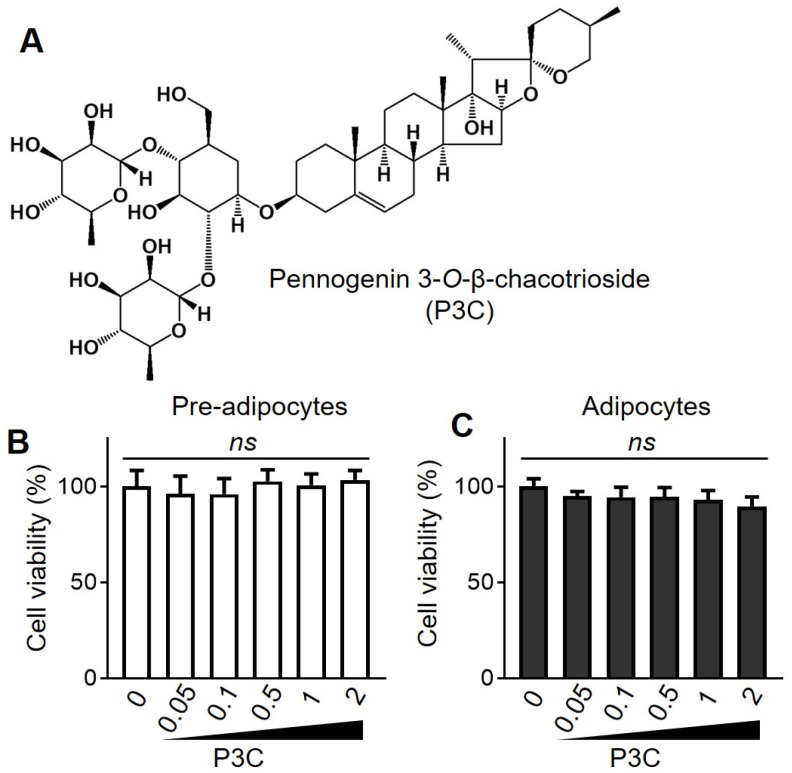
**Effects of P3C on viability of 3T3-L1 adipocytes.** (**A**) Chemical structure of P3C. (**B**) 3T3-L1 pre-adipocytes were treated with P3C (0–2 μM). (**C**) 3T3-L1 pre-adipocytes were differentiated in a medium containing MDI in the presence of P3C (0–2 μM) for 8 d. Cell viability was measured using a WST-1 kit. Data are presented as the mean ± standard deviation (SD; n ≥ 5). Statistical significance (*p* value) was determined using Tukey’s post hoc test. ns, not significant.

We performed a cell viability assay to assess the cytotoxicity of P3C (Figure 1A), at concentrations ranging from 0.05 to 2 µM, on pre-adipocytes and mature adipocytes. Compared to the DMSO-treated group, P3C exerted no cytotoxic effects on either pre-adipocytes or mature adipocytes at concentrations ≤ 2 µM, as shown in Figure 1B. Subsequently, we investigated the effects of P3C, at 0.5 or 1 μM, on lipid accumulation during adipocyte differentiation.

### 2.1. P3C Decreased Lipid Accumulation in Hypertrophied 3T3-L1 Adipocytes

**Figure 2:**
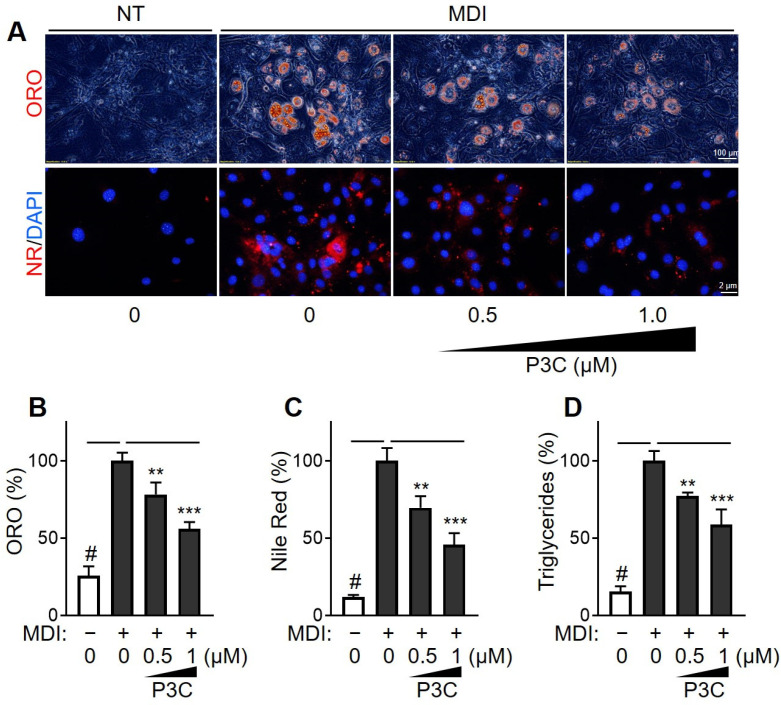
**Effects of P3C on lipid accumulation in hypertrophied 3T3-L1 adipocytes.** 3T3-L1 cells were seeded and induced to differentiate in the presence of P3C (0.5 or 1.0 µM) for 8 d. (**A**) Cells were stained with Oil Red O (ORO) or Nile red (NR) solution to determine the accumulation of lipid droplets. NT, not treated. DAPI = nuclei. (**B**) ORO-stained cells were extracted using isopropanol, and lipid content was quantified at 550 nm using a spectrophotometer. (**C**) NR-stained cells were monitored using a fluorescence microscope and quantified using a fluorescent reader (Ex/Em = 555/630 nm). (**D**) Total TG content was measured using a TG assay kit. Data are presented as the mean ± standard deviation (SD; n ≥ 4). # *p* < 0.0001 versus the undifferentiated group; ** *p* < 0.01; *** *p* < 0.0001 versus the P3C-treated group, as determined using a one-way ANOVA followed by Tukey’s post hoc test.

Adipocyte hypertrophy results from a continuous energy supply and insulin receptor signaling, leading to triglyceride (TG) accumulation. Consequently, we evaluated the effects of P3C on lipid deposition in mature 3T3-L1 adipocytes using Oil Red O (ORO) and Nile red (NR) staining (Figure 2A–C). Treatment with the differentiation-inducing mixture (MDI) significantly increased the rate of formation of lipid droplets, as confirmed by the intensified staining with both ORO and NR, indicating an enhanced lipid accumulation during differentiation. Notably, the increase in lipid droplet formation following differentiation was dose-dependently reduced by P3C treatment. Furthermore, compared to the DMSO-treated group, the P3C treatment dose-dependently decreased the elevated TG levels in differentiated 3T3-L1 cells (Figure 2D). These findings demonstrate the effective disruption of lipid accumulation by P3C in 3T3-L1 cells during differentiation.

### 2.2. P3C Decreased the Expression of Lipogenesis-Related Genes in Hypertrophied 3T3-L1 Adipocytes

**Figure 3:**
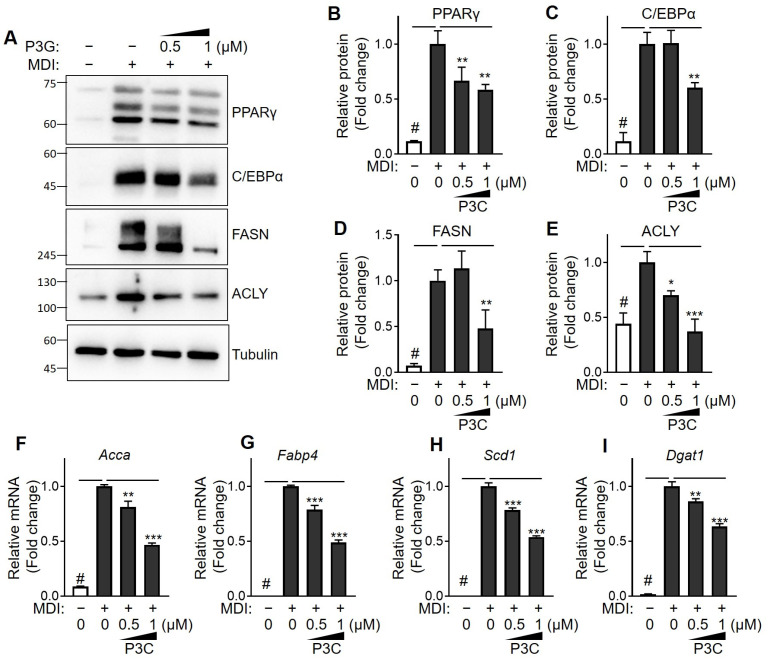
**Effects of P3C on the expression of lipid-metabolism-related proteins and key regulators in hypertrophied 3T3-L1 adipocytes.** 3T3-L1 cells were seeded and induced to differentiate in the presence of P3C (0.5 or 1.0 µM) for 8 d. (**A**) Protein expression of PPARγ, C/EBPα, FASN, and ACLY was analyzed using immunoblotting analysis. (**B**–**E**) Bar graphs indicate the densitometric quantification of PPARγ/tubulin, C/EBPα/tubulin, FASN/tubulin, and ACLY/tubulin bands. Data are expressed as the mean ± standard deviation (SD; n = 3). (**F**–**I**) mRNA expression of genes related to lipogenesis in 3T3-L1 adipocytes was analyzed using RT-qPCR. Data are presented as the mean ± standard deviation (SD; n ≥ 3). # *p* < 0.0001 versus the undifferentiated group. * *p* < 0.05; ** *p* < 0.01; *** *p* < 0.0001 versus the P3C-treated group, as determined using a one-way ANOVA followed by Tukey’s post hoc test.

To further elucidate the anti-lipid accumulation effects of P3C in 3T3-L1 cells and to understand the mechanism underlying its inhibition of adipogenesis, we evaluated the protein expressions of PPARγ, C/EBPα, FASN, and ACLY (Figure 3A–E). Following differentiation, the expression of these proteins exhibited a noticeable increase in lipid accumulation and P3C significantly mitigated this increase. Additionally, mRNA analysis revealed a significant upregulation of the expression of lipogenesis-related genes, such as *Acca*, *Fabp4*, *Scd1*, and *Dgat1*, during differentiation compared to those in the DMSO-treated group (Figure 3F–I). However, treatment with P3C effectively and dose-dependently suppressed the upregulated expression of these genes. These findings indicate that P3C ameliorates lipid accumulation by inhibiting the expression of adipogenic and lipogenic genes during adipocyte differentiation.

### 2.3. P3C Increased the Expression of Fatty Acid Oxidation-Related Genes in Hypertrophied 3T3-L1 Adipocytes

**Figure 4:**
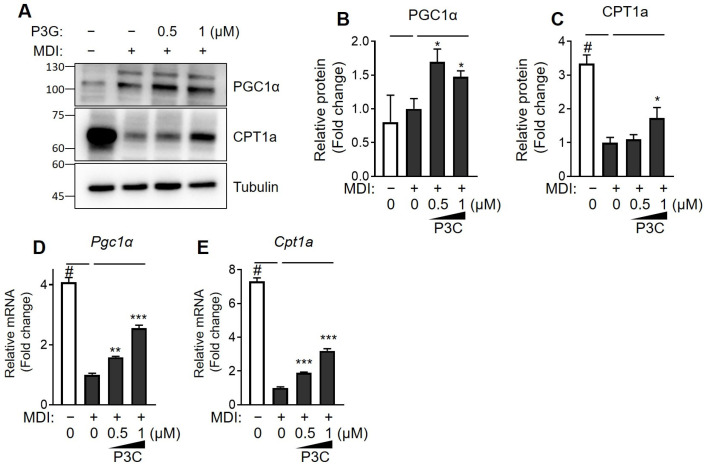
**Effects of P3C on the expression of fatty acid oxidation-related genes in hypertrophied 3T3-L1 adipocytes.** 3T3-L1 cells were seeded and induced to differentiate in the presence of P3C (0.5 or 1.0 µM) for 8 d. (**A**) Protein expression of PGC1α and CTP1a was determined using immunoblotting. (**B**,**C**) The bar graph indicates the relative intensity of PGC1α/tubulin and CPT1a/tubulin bands. mRNA expression levels of (**D**) *Pgc1α* and (**E**) *Cpt1a* were analyzed using RT-qPCR. Data are presented as the mean ± standard deviation (SD; n ≥ 3). # *p* < 0.0001 versus the undifferentiated group. * *p* < 0.05; ** *p* < 0.01; *** *p* < 0.0001 versus the P3C-treated group, as determined using a one-way ANOVA followed by Tukey’s post hoc test.

To assess whether P3C influenced fatty acid oxidation (FAO), we examined changes in the protein and mRNA expression of PGC1α and CPT1a. Following P3C treatment, there was a notable increase in the protein expression of PGC1α and CPT1a in comparison to the DMSO-treated group (Figure 4A–C). Additionally, the mRNA expression of *Pgc1α* and *Cpt1a* exhibited a significant dose-dependent increase after P3C treatment (Figure 4D,E). These findings suggest that P3C suppresses the expression of adipogenic and lipogenic factors by enhancing FAO.

### 2.4. P3C Enhanced the Mitochondrial Oxidative Capacity in Hypertrophied 3T3-L1 Adipocytes

**Figure 5:**
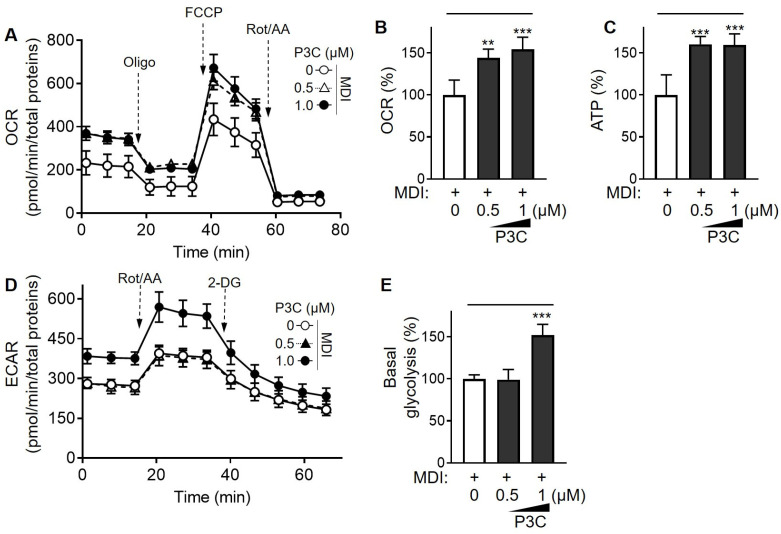
**Effects of P3C on mitochondrial oxidative function in hypertrophied 3T3-L1 adipocytes.** 3T3-L1 cells were seeded and induced to differentiate in the presence of P3C (0.5 or 1.0 µM) for 8 d. (**A**,**B**) OCR and (**C**) ATP generation were measured. (**D**) Extracellular acidification rate (ECAR) and (**E**) basal glycolysis were measured. Representative data from three independent experiments are presented. The indicated values were normalized to the protein content determined using a BCA assay. Data are presented as the mean ± standard deviation (SD; n ≥ 4). ** *p* < 0.01; *** *p* < 0.0001 versus the P3C-treated group, as determined using a one-way ANOVA followed by Tukey’s post hoc test.

**Figure 6:**
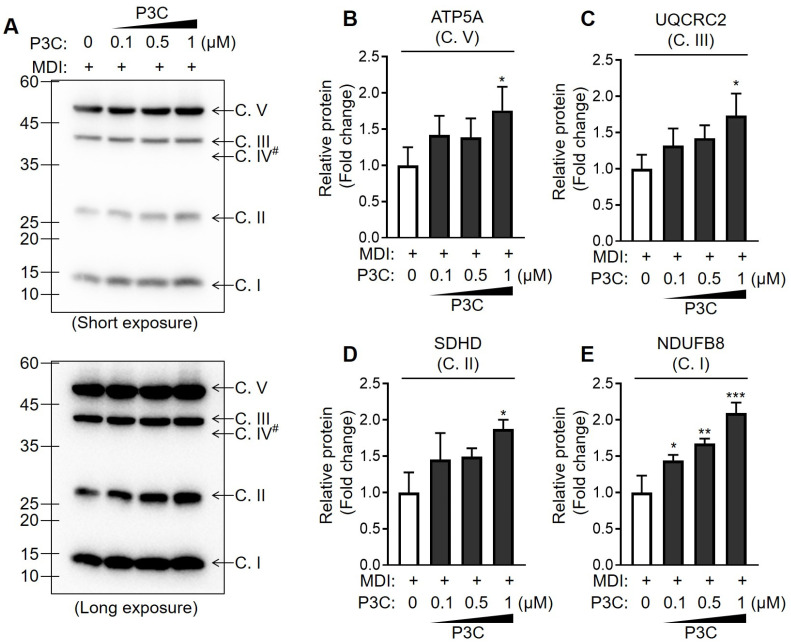
**Effects of P3C on the expression of OXPHOS complex proteins in hypertrophied 3T3-L1 adipocytes.** 3T3-L1 cells were seeded and induced to differentiate in the presence of P3C (0.5 or 1.0 µM) for 8 d. (**A**–**E**) Mitochondrial fraction was isolated. Subunits of the OXPHOS complex were separated through SDS-PAGE, analyzed using immunoblotting, and were subjected to the densitometric quantification of each band from the OXPHOS complex. (**B**) ATP synthase subunit H, ATP5A; (**C**) ubiquinol-cytochrome C reductase core protein 2, UQCRC2; (**D**) succinate dehydrogenase complex subunit D, SDHD; (**E**) NADH: ubiquinone oxidoreductase subunit B8, NDUFB8. Data are presented as the mean ± standard deviation (SD; n ≥ 3). #, the Complex IV subunit (with a theoretical molecular weight of 38 kD) was not detected. * *p* < 0.05; ** *p* < 0.01; *** *p* < 0.0001 versus the P3C-treated group, as determined using a one-way ANOVA followed by Tukey’s post hoc test.

The phenotypic mitochondrial energy metabolism plays a pivotal role in shaping the function and integrity of adipocytes [27]. Therefore, we investigated the alterations in mitochondrial respiration activity through real-time metabolic analysis in differentiated 3T3-L1 cells following P3C treatment. Notably, in differentiated adipocytes, the P3C-treated group exhibited a significant increase in cellular oxygen consumption rate (OCR) levels compared to that in the DMSO-treated group (Figure 5A,B). Furthermore, P3C treatment led to a notable increase in intracellular ATP production (Figure 5C). Additionally, the extracellular acidification rate (ECAR) was increased in P3C-treated differentiated adipocytes compared to those treated with DMSO (Figure 5D,E). To further investigate the effect of P3C on mitochondrial integrity, we assessed alterations in mitochondrial contents and membrane potential using qPCR analysis and a mitochondrial staining assay, respectively. Specifically, P3C treatment increased mitochondrial mass and DNA content in differentiated adipocytes (Figures S1 and S2). Moreover, Mito Red staining of the P3C-treated group was more intense than that of the DMSO-treated group, indicating an intensification of mitochondrial membrane potentials (Figure S2). Subsequently, we isolated mitochondrial fractions to examine the protein expression of the OXPHOS complex. Treatment with P3C significantly upregulated the expression levels of mitochondrial OXPHOS complex proteins in a dose-dependent manner and (Figure 6 and Figure S3). Taken together, our findings suggest that P3C has the capability to enhance mitochondrial membrane integrity, thereby contributing to the improvement in mitochondrial oxidative capacity in hypertrophied adipocytes.

## 3. Discussion

The present study clearly demonstrates that P3C treatment significantly suppresses lipid accumulation during adipocyte differentiation without inducing cytotoxicity. This study provides the first documented evidence of the specific antilipidemic mechanisms mediated by P3C in differentiated 3T3-L1 cells.

Notably, P3C reduced the expression of adipogenic/lipogenic genes at the protein and mRNA levels. The P3C-mediated downregulation of transcriptional factors, including PPARγ and C/EBPα, could induce an overall decrease in the expression of genes related to lipogenesis. It is well described that fat accumulation in the adipocytes is governed by the interplay of transcriptional factors [28]. PPARγ serves as a master regulator in adipogenesis, facilitated by its collaboration with C/EBPα [29]. The crosstalk between PPARγ and C/EBPα regulates the downstream genes associated with a range of signaling pathways for lipid synthesis and accumulation [30]. Additionally, data from direct chromatin immunoprecipitation analysis showed that PPARγ and C/EBPα share the promoter region for their lipogenic target genes [31]. Thus, our data imply that P3C may inhibit lipid accumulation in adipocytes through direct regulation at the transcriptional level.

PGC1α is a transcriptional coactivator that plays a crucial role in regulating various metabolic processes, including the breakdown of fatty acids in lipid metabolism [32]. Functioning as a master regulator of mitochondrial biogenesis and function, PGC1α stimulates the expression of genes, including *CPT1a*, associated with mitochondrial long-chain FAO [33]. CPT1a is an enzyme located on the outer mitochondrial membrane and serves as a key regulator of FAO [34]. CPT1a enables the transport of long-chain fatty acids into the mitochondria, where they can undergo beta-oxidation for energy production [34]. Therefore, PGC1α and CPT1a collaborate effectively during heightened cellular energy demand [35]. Our study showed that P3C treatment increased the protein and mRNA expression of PGC1α and CPT1a in hypertrophied 3T3-L1 adipocytes. Notably, the downregulation of PGC1α expression has been implicated in the development and progression of metabolic disorders, including obesity and insulin resistance [36,37]. Phytochemicals have the potential to offer beneficial effects against obesity by enhancing the PGC1α pathway [38,39,40].

PGC1α plays a central role in coordinating the expression of the genes involved in mitochondrial biogenesis and function, including those related to the tricarboxylic acid cycle. Accordingly, the upregulation of PGC1α results in an increase in the oxidative capacity of cells by upregulating mitochondrial oxygen respiration. Therefore, we assessed alterations in mitochondrial respiration after treatment with P3C in differentiated 3T3-L1 cells to evaluate the impact of P3C on the condition of over-accumulated lipids. Our data indicate that P3C treatment can improve mitochondrial oxidative function by increasing OXPHOS complex protein levels and intracellular ATP production, possibly resulting from an increased mitochondrial membrane integrity. Consistent with our results, sinapic acid, a plant-derived phenolic compound, upregulated PGC1α expression and promoted OXPHOS protein levels in 3T3-L1 adipocytes [41]. Additionally, an increase in ATP and OCR was observed with the treatment of isoorientin, a *C*-glycosyl flavonoid, leading to a decrease in lipid accumulation in differentiated adipocytes [42]. Moreover, salvianolic acid B can alleviate lipid overload by enhancing PGC1α-mediated mitochondrial oxidative function [43]. Therefore, our study provides evidence that P3C treatment positively influences the mitochondrial energy metabolism of adipocytes, contributing to the suppression of lipid accumulation. 

Mitochondria contain multiple copies of circular DNA that encode 13 electron transport chain (ETC) subunits [44]. Despite mtDNA only encoding 13 proteins, they play a pivotal role in sustaining mitochondrial functions, particularly OXPHOS [45]. During nutrition overload, mitochondrial integrity is often compromised due to dysregulation in the transcription and translation of mtDNA, leading to the accumulation of dysfunctional mitochondrial proteins [46]. Consequently, disruption in the quality control of mitochondrial proteins results in bioenergetic and biosynthetic malfunctions, contributing to severe metabolic disorders, including obesity [27]. Notably, mitochondrial respiration is downregulated in the adipose tissue of obese individuals, and this mitochondrial dysfunction is closely associated with progression to the chronic state of diseases [47]. Thus, maintaining adequate mitochondrial DNA content and protein quality is crucial in the development and progression of obesity and associated diseases [48]. Our study highlights that P3C treatment not only increased mtDNA mass but also elevated the expression of mitochondrial ETC proteins in differentiated adipocytes. P3C-treated differentiated adipocytes exhibited significantly enhanced mitochondrial respiratory activity and membrane potential, suggesting that P3C may improve mitochondrial protein quality to enhance its oxidative capacity. These findings underscore the positive impact of P3C on mitochondrial bioenergetics, including OXPHOS, and propose its potential to ameliorate lipid metabolism, thereby offering insights into the development of strategies to mitigate the development and progression of obesity.

P3C is classified structurally as a steroidal saponin, consisting of a hydrophilic glycoside moiety attached to a lipophilic steroidal group (as the aglycone). Studies have suggested that pennogenin glycosides exhibit higher anti-inflammatory activity than their aglycone forms [49]. Furthermore, structure-activity assays have demonstrated the crucial role of the glycoside moiety in the anti-tumor activity of pennogenin steroidal saponins against human liver cancer [50]. On the other hand, both the aglycone and sugar moieties of pennogenin glycosides are essential for their pharmacologic actions on hemostasis [51]. Additionally, pennogenin (aglycone moiety) has been reported to exhibit a significant suppressive activity on oxidative stress [52]. Although further studies are required to identify the specific molecule responsible for the antilipidemic activity of P3C, both the glycoside moiety and aglycone of P3C may be crucial for its anti-lipid accumulation effects. Additionally, the specific mechanism by which P3C downregulates the expression of PPARγ and C/EBPα, thereby mediating adipogenesis, remains unclear. Mitochondrial protein quality control is strongly associated with the mechanisms of mitochondrial maintenance, including mitochondrial dynamics (fusion and fission) and mitophagy, which are implicated in the development and progression of metabolic disorders, including obesity. Therefore, further research is needed to elucidate the effects of P3C on mitochondrial protein quality control in adipocytes. Moreover, our investigation focused solely on the antilipidemic effects of P3C at the cellular level. Consequently, additional animal studies are necessary to explore the potential pharmaceutical benefits of P3C.

## 4. Materials and Methods

### 4.1. Chemicals and Reagents

P3C was purchased from MedChem Express (#HY-N4180, Monmouth Junction, NJ, USA). Dimethyl sulfoxide (DMSO, #276855), Oil Red O (ORO, #O1516), Nile red (NR, #N3013), 4′,6-diamidino-2-phenylindole dihydrochloride, 2-(4-amidinophenyl)-6-indolecarbamidine dihydrochloride (DAPI, #9542), isobutylmethylxanthine (IBMX, #I5879), dexamethasone (Dex, #D1756), insulin (Ins, #I6634), and other reagents were acquired from Sigma-Aldrich (St. Louis, MO, USA). Antibodies against PPARγ (16643-1-AP, Proteintech, Chicago, IL, USA), C/EBPα (#2295, Cell Signaling Technology, Danvers, MA, USA), fatty acid synthase (FASN, #sc-48357, Santa Cruz Biotechnology, Santa Cruz, CA, USA), ATP-citrate lyase (ACLY, #15421-1-AP, Proteintech), peroxisome proliferator-activated receptor gamma coactivator 1-α (PGC1α; #66369-1-Ig, Proteintech), carnitine palmitoyltransferase 1A (CPT1a; #15184-1-AP, Proteintech), total OXPHOS cocktail (#ab110413, Abcam, Cambridge, MA, USA), and α/β-tubulin (#2148, Cell Signaling Technology) were used for Western blotting.

### 4.2. Cell Culture

Mouse 3T3-L1 pre-adipocytes were obtained from the American Type Culture Collection (Manassas, VA, USA). The cells were cultured in high-glucose Dulbecco’s Modified Eagle Medium (DMEM, Welgene, Gyeongsan, Republic of Korea), supplemented with 10% bovine calf serum (BCS, Welgene) and a 1% penicillin–streptomycin solution (10,000 U/mL; Gibco, Carlsbad, CA, USA), and maintained at 37 °C in a 5% CO_2_ atmosphere. To induce differentiation, confluent cells were incubated at 37 °C in DMEM containing 10% fetal bovine serum (FBS, Welgene) and MDI composed of 0.5 mM IBMX, 1 µM Dex, and 10 µg/mL Ins for 48 h. The culture medium was subsequently refreshed with DMEM containing 10% FBS and 10 µg/mL Ins every other day. During the adipogenesis process, the cells were either treated with P3C or DMSO (control group) for 8 d.

### 4.3. Cell Viability Assay

Cytotoxicity of P3C was assessed using the water-soluble tetrazolium salt-1 (WST-1) assay (#11644807001, Roche, Mannheim, Germany). In brief, 3T3-L1 cells were seeded in 96-well plates (2 × 10^4^ cells per well) containing DMEM supplemented with 10% BCS. Subsequently, P3C was added to each well, and the cells were cultured for 48 h. To evaluate the cytotoxicity of P3C on mature adipocytes, 3T3-L1 cells were allowed to differentiate into mature adipocytes, followed by treatment with various concentrations of P3C every 2 d. The culture medium was replaced with fresh medium containing Ins. Thereafter, 10% of WST-1 solution was added to each well, and the cells were incubated for an additional 3 h at 37 °C in a 5% CO_2_ atmosphere. The conversion of WST-1 to formazan by mitochondrial dehydrogenases in the supernatant was quantified spectrophotometrically at 450 nm using a microplate reader (Molecular Devices, Sunnyvale, CA, USA).

### 4.4. Quantification of Lipid Accumulation

Lipid droplet formation in adipocytes was determined using ORO and NR staining. For ORO staining, cells were seeded in 24-well plates at a density of 5 × 10^4^ cells per well and allowed to differentiate. Differentiated cells were washed twice with Dulbecco’s phosphate-buffered saline (DPBS, Welgene) and fixed with a 3.7% formaldehyde solution (Biosesang, Yongin, Republic of Korea) for 15 min at 25 °C. After washing with 60% isopropanol (Ducksan, Ansan, Republic of Korea), the cells were stained with ORO solution (Sigma-Aldrich; 0.3% in 60% propylene glycol) for 20 min at 25 °C. After removing the staining solution, the plate was washed with distilled water and dried, and cellular lipid droplets were imaged using an Olympus IX73 light microscope (Olympus, Center Valley, PA, USA). Following microscopic observation, the ORO stain was eluted with 100% isopropanol and the absorbance was measured at 505 nm using a microplate reader (Molecular Devices).

For NR staining, cells (1 × 10^4^ cells) in 8-well chamber slides were seeded and allowed to differentiate. Before fixation, cells were stained with NR (1 μM) for 30 min. The stained cells were fixed using a 3.7% formaldehyde solution. After washing with DPBS, the nuclei were stained with DAPI (100 ng/mL in PBS) for 2 min. The stained lipid droplets were visualized using a fluorescence microscope (Olympus DP71). To quantify the lipid content, the cells were dried, isopropanol was added, and fluorescence was analyzed using a microplate fluorescent reader (Ex/Em = 555/630 nm; Molecular Devices).

### 4.5. Quantification of Total TG

Total TG was quantified using a TG assay kit (#ab65336, Abcam), following the manufacturer’s protocols. After removing the medium, cells were washed with DPBS and placed in a 0.5% NP-40 solution. Subsequently, cell suspensions were homogenized and solubilized by heating at 80 °C and then cooled to 24 °C for three cycles. TG was enzymatically converted into glycerol and fatty acids through lipase treatment. The samples were then centrifuged at 15,000× *g* for 3 min to collect soluble TG, and the absorbance was measured at 570 nm using a microplate reader (Molecular Devices). Individual values were normalized to the total protein concentration using the bicinchoninic acid (BCA) kit (#BCA1, Sigma-Aldrich). 

### 4.6. Western Blotting Analysis

Cells were washed with cold PBS and lysed in RIPA buffer (#R0278, Sigma-Aldrich) containing protease and phosphatase inhibitor cocktails (Roche). The total protein concentration in the lysates was determined using the BCA kit (#BCA1, Sigma-Aldrich). The proteins were mixed with 5X SDS-PAGE buffer (Biosesang), separated using SDS-PAGE (8% or 12%), and then transferred to nitrocellulose or PVDF membranes (Bio-Rad, Hercules, CA, USA) at 70 V for 60 min. The membranes were blocked with 5% non-fat dry milk in Tris-buffered saline containing Tween-20 (TBST) for 1 h at 4 °C to prevent nonspecific binding. Subsequently, the membranes were incubated overnight at 4 °C with primary antibodies diluted at 1:500 or 1:1000. After washing with TBST, the membranes were incubated with the appropriate secondary antibodies (Sigma-Aldrich) for 1 h at 25 °C. The immunoblots were developed using a chemiluminescent substrate (Thermo Fisher Scientific, Sunnyvale, CA, USA). Protein expression was then quantified using ImageJ (version 1.54d, NIH, Bethesda, MD, USA). 

### 4.7. Quantitative Real-Time PCR

Total RNA was extracted using the RNeasy mini kit (Qiagen, Hilden, Germany). cDNA was synthesized using a cDNA reverse transcription kit (#FSQ-301, TOYOBO Co., Ltd., Osaka, Japan). To verify the quality of the extracted RNA, we measured the absorbance at wavelengths of 260 nm for RNA concentration and 280 nm for protein contamination. This provided an estimate of RNA purity via the A260/A280 ratio, with a target ratio close to 2.0 indicating pure RNA. The real-time polymerase chain reaction (PCR) analysis was performed using Faststart Universal SYBR Green Master (#4913914001, Roche) and specific primers. The primer sequences used for RT-qPCR are listed in Table S1. The relative mRNA levels of target genes were normalized to those of the housekeeping genes (RPLP0 or GAPDH) and calculated using the ΔΔCt method.

### 4.8. Measurement of Oxygen Consumption Rate 

For the analysis of mitochondrial metabolism, 3T3-L1 cells were plated in Seahorse XF cell culture plates (Agilent Technologies, Palo Alto, CA, USA) at a density of 10,000 cells per well. Cellular differentiation was induced as previously described in a prior study [53], and before measurements, the culture medium was replaced with an identical nutrient composition medium devoid of buffering agent (#103575-100, Agilent Technologies). After a 1 h incubation in a non-CO_2_ incubator for degassing, the real-time oxygen consumption rate (OCR) was measured using a Seahorse XF analyzer (Agilent Technologies). Subsequently, cells were sequentially exposed to 1.5 μM of oligomycin, 1 μM of carbonyl cyanide 4-(trifluoromethoxy) phenylhydrazone (FCCP), and 1 μM of rotenone/antimycin A mixture. The OCR was monitored three times both before and after the injection of the aforementioned reagents. Mitochondrial respiration was determined by calculating the differences between the maximal OCR following FCCP injection and basal OCR. ATP production was assessed by subtracting the non-mitochondrial OCR observed after oligomycin injection from the basal OCR. The mitochondrial respiration data were adjusted to the values of the control group.

### 4.9. Glycolytic Rate Analysis

For glycolytic rate analysis in fully differentiated 3T3-L1 cells, the medium was replaced with a non-buffered basal medium (Agilent Technologies), supplemented with 25 mM of glucose, 1 mM of sodium pyruvate, 4 mM of glutamine, and 0.5 mM HEPES, at pH 7.4. Thereafter, cells were incubated at 37 °C in a non-CO_2_ incubator for 1 h for degassing. Then, the extracellular acidification rate (ECAR) was measured using the Seahorse XF analyzer (Agilent Technologies) in a cycle with 6 min intervals (mixing and measuring) at the basal conditions and followed by oligomycin (5 μM) and 2-deoxyglucose (2-DG; 50 mM) injections. The ECAR and basal glycolysis were calculated as previously described [54]. Following calculations, the basal and compensatory glycolysis data were normalized relative to the group.

### 4.10. Measurement of Mitochondrial DNA Levels

DNA isolation from the 3T3-L1 cells was carried out using a DNA purification kit from BIONEER Corp. (Daejeon, Republic of Korea). The quantification of mitochondrial DNA (mtDNA) relative to genomic DNA was performed using the qPCR method. RT-qPCR primers used in this analysis are detailed in Table S1. The average mtDNA/nuclear(n)DNA ratio for each sample was calculated using the ΔΔCT method.

### 4.11. Mitochondrial Staining Assay 

The cells were fixed with a 3.7% formaldehyde solution. After washing with PBS, fixed cells were incubated with 500 nM of MitoTracker^TM^ Red CMXRos (Invitrogen, Waltham, MA, USA) in PBS for 30 min at 37 °C. After rinsing with PBS thrice, cell nuclei were stained with 4’,6-diamidino-2-phenylindole (DAPI) for 5 min, and fluorescence images of the cells were captured using an Olympus DP71 fluorescence microscope (Olympus, Tokyo, Japan). The fluorescence intensity was measured in randomly selected regions and quantitatively analyzed using ImageJ software (version 1.54d).

### 4.12. Mitochondria Isolation

Mitochondrial fractions were obtained from 3T3-L1 adipocytes using a mitochondria isolation kit (#ab65320, Abcam) following the manufacturer’s instructions. Cells were lysed with cytosol isolation buffer containing a protease inhibitor cocktail, disrupted by 70 strokes in a Dounce homogenizer. Cell debris and the nuclear fraction were subsequently removed by centrifugation at 700× *g* for 10 min, and mitochondrial fractions were precipitated via centrifugation at 10,000× *g* for 25 min. To achieve highly enriched mitochondrial fractions, samples were washed with mitochondrial isolation buffer, and the final pellet obtained after centrifugation was utilized as the mitochondrial fractions.

### 4.13. Statistical Analysis 

Data are presented as the mean ± standard error of the mean of at least three replications. Statistical significance was determined using a one-way analysis of variance (ANOVA), followed by Tukey’s post hoc test for multiple comparisons or unpaired two-tailed Student’s *t*-test in Prism 8 software (GraphPad Software, San Diego, CA, USA). Statistical significance was set at *p* < 0.05.

## 5. Conclusions

In conclusion, we demonstrated the ability of P3C to suppress the expression of lipogenic factors and enhance mitochondrial oxidative capacity in hypertrophied adipocytes, thus presenting a valuable strategy for preventing and treating excessive lipid accumulation. This study underscores the potential of P3C as a promising candidate for further investigation and development as a therapeutic agent for obesity-related diseases. 

## Data Availability

The data presented in this study are available upon request from the corresponding author.

## Supplementary Materials

The supporting information can be downloaded at: https://www.mdpi.com/article/10.3390/ijms25052970/s1.
