# Supplementary material for: Pennogenin 3-O-β-Chacotrioside Attenuates Hypertrophied Lipid Accumulation by Enhancing Mitochondrial Oxidative Capacity
Source: Int J Mol Sci. 2024 Mar 4;25(5):2970. doi: 10.3390/ijms25052970 (PMC10931814; doi:10.3390/ijms25052970)
Supplement: Supplementary file 1 [file ijms-25-02970-s001.zip › Figures S1-S3.pdf]

Supplementary Material

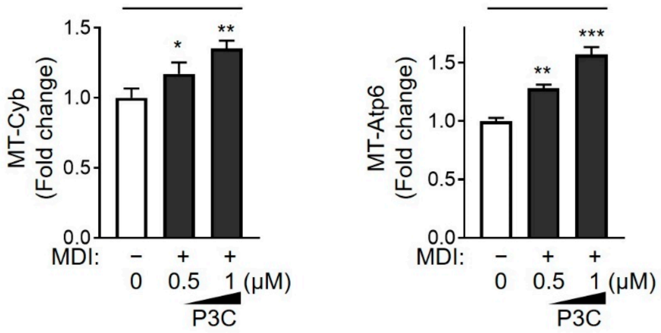

Figure S1. Effects of P3C on mtDNA in hypertrophied 3T3-L1 adipocytes. 3T3-L1 cells were seeded and induced to differentiate in the presence of P3C (0.5 or 1.0 μM) for 8 d. Relative mtDNA copy number was measured using RT-qPCR to assess the relative mitochondrial (Mito) DNA amount. Data are presented as the mean ± standard deviation (SD; n ≥ 4). *$p < 0.05$; **$p < 0.001$; ***$p < 0.0001$ versus the P3C-treated group, as determined by one-way ANOVA followed by Tukey's post hoc test.

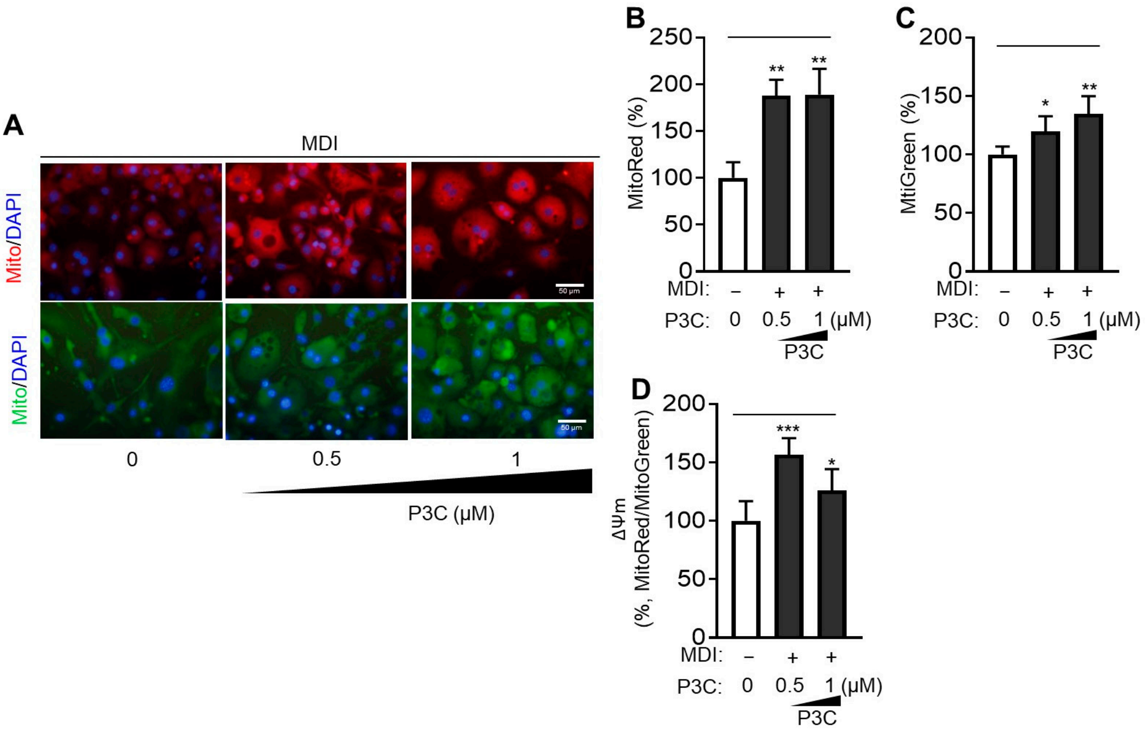

Figure S2. Effects of P3C on mitochondrial membrane potential in hypertrophied 3T3-L1 adipocytes. 3T3-L1 cells were seeded and induced to differentiate in the presence of P3C (0.5 or 1.0 μM) for 8 d. Representative images of Mito Tracker Red-stained adipocytes and MitoTracker Green-stained adipocytes are shown. (**A**) Representative images of CMXRos-stained adipocytes (for mitochondrial membrane potential, ΔΨm, top) and MitoGreen-stained adipocyte (for mitochondrial mass, bottom) are displayed. (**B, C**) Quantitative analysis of fluorescence intensity (SD; n ≥ 5) is shown. (D) Relative mitochondrial membrane potential (ΔΨm) was determined by assessing the ratio of MitoRed to MitoGreen intensity. *$p < 0.05$, **$p < 0.001$, ***$p < 0.0001$ versus the P3C-treated group, as determined by one-way ANOVA followed by Tukey's post hoc test.

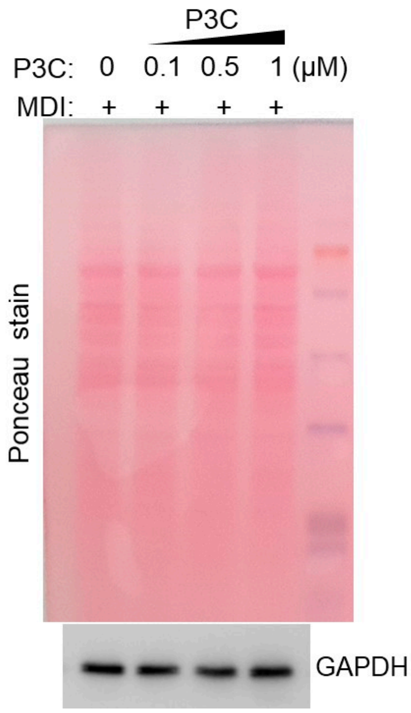

Figure S3. Effects of P3C on OXPHOS complex proteins in hypertrophied 3T3-L1 adipocytes. Protein from the indicated cell lysates were analyzed by western blotting. The Ponceau stain of the PVDF is presented to confirm equal protein loading. Protein expression of GAPDH, determined by immunoblotting, was used as the house keeping gene.
