# Supplementary material for: Pennogenin 3-O-β-Chacotrioside Attenuates Hypertrophied Lipid Accumulation by Enhancing Mitochondrial Oxidative Capacity
Source: Int J Mol Sci. 2024 Mar 4;25(5):2970. doi: 10.3390/ijms25052970 (PMC10931814; doi:10.3390/ijms25052970)
Supplement: Supplementary file 1 [file ijms-25-02970-s001.zip › Table S1.pdf]

Table S1. Oligonucleotide sequences used in RT-PCR analysis.

Target gene	Direction	Sequence (5′-3′)	Annealing temperatures	GenBank accession
Pparγ	forward<br>reverse	GCGGGCTGAGAAGTCACGTT<br>CCATCACGGAGAGGTCCACA	58 °C	NM_001127330.3
C/ebpα	forward<br>reverse	TACCGAGTAGGGGGAGCA<br>TCATTTTTCTCACGGGGCC	55 °C	NM_001287514.1
Fasn	forward<br>reverse	AGAAGCCATGTGGGGAAGATT<br>AGCAGGGACAGGACAAGACAA	56 °C	NM_007988.3
Acly	forward<br>reverse	CTTGGGCCGGAACAAAA<br>GCCGAGGTGGTGCAGAT	55 °C	NM_001199296.1
Acca	forward<br>reverse	AGAAACCCGAACAGTGGAACT<br>AGGTAGCCCTTCACGGTTAAA	55 °C	NM_133360.3
Fabp4	forward<br>reverse	GGGAACCTGGAAGCTTGTCT<br>ACTCTCTGACCGGATGGTGA	55 °C	NM_001409514.1
Scd1	forward<br>reverse	TTCTTGCGATACACTCTGGTGC<br>CGGGATTGAATGTTCTTGTCGT	55 °C	NM_009127.4
Dgat1	forward<br>reverse	GGCCCAAGGTAGAAGAGGAC<br>GATCAGCATCACCACACACC	55 °C	NM_010046.4
Pgc1α	forward<br>reverse	AAGTGGTGTAGCGACCAATCG<br>AATGAGGGCAATCCGTCTTCA	55 °C	NM_008904.3
Cpt1a	forward<br>reverse	CTCCGCCTGAGCCATGAAG<br>CACCAGTGATGCCATTCT	55 °C	NM_013495.2
Rplp0	forward<br>reverse	AGGTCCTCCTTGGTGAAC<br>GTGCTGATGGGCAAGAAC	52 °C	NM_007475.5
Gapdh	forward<br>reverse	AGTATGACTCCACTCACGGCAAAT<br>GTCTCGCTCCTGGAAGATGGT	56 °C	NM_001411843.1
nDNA H19	forward<br>reverse	GAACAGAAGCATTCTAGGCTGG<br>TTCTAAGTGAATTACGGTGGGTG	55 °C	NR_130974.1
MT-Cyb	forward<br>reverse	ACGCAAACGGAGCCTCAATA<br>TGTGGCTATGACTGCGAACA	55 °C	NC_005089.13
MT-Atp6	forward<br>reverse	CCAATGGCATTAGCAGTCCG<br>AATGGTAGCTGTTGGTGGGC	55 °C	NC_005089.1

Note: peroxisome proliferator-activated receptor-γ, Ppar-γ; CCAAT/enhancer-binding protein α, C/ebpα; fatty acid synthase, Fasn; ATP-citrate lyase, Acly; acetyl-coA Carboxylase α, Acca; fatty acid-binding protein 4, Fabp4; stearoyl-Coenzyme A desaturase 1, Scd1; diacylglycerol O-acyltransferase 1, Dgat1; Peroxisome proliferator-activated receptor γ coactivator 1 α, Pgc1α; carnitine palmitoyltransferase 1A, Cpt1a; Ribosomal protein lateral stalk subunit P0, Rplp0; Glyceraldehyde-3-phosphate dehydrogenase, Gapdh. Nuclear DNA H19, nDNA H19; Mitochondrially Encoded Cytochrome B, MT-Cyb; Mitochondrially Encoded ATP Synthase Membrane Subunit 6, MT-Atp6.
